# How common is remission in rheumatoid factor-positive juvenile idiopathic arthritis patients? The multicenter Pediatric Rheumatology Academy (PeRA) research group experience

**DOI:** 10.1186/s12969-023-00860-5

**Published:** 2023-07-20

**Authors:** Semanur Ozdel, Hafize Emine Sönmez, Şengül Çağlayan, Özlem Akgün, Tuncay Aydın, Özge Baba, İlknur Bağrul, Gülçin Otar Yener, Kübra Öztürk, Ferhat Demir, Deniz Gezgin Yıldırım, Şerife Gül Karadağ, Esra Bağlan, Mustafa Çakan, Mukaddes Kalyoncu, Balahan Bora Makay, Şevket Erbil Ünsal, Sevcan Bakkaloğlu, Mehmet Bülbül, Betül Sözeri, Nuray Aktay Ayaz

**Affiliations:** 1 Department of Pediatric Rheumatology, Dr Sami Ulus Maternity and Child Health and Diseases Training and Research Hospital, Ankara, Turkey; 2 Department of Pediatric Rheumatology, Etlik City Hospital, Ankara, Turkey; 3 Department of Pediatric Rheumatology, Faculty of Medicine, Kocaeli University, Kocaeli, Turkey; 4 Department of Pediatric Rheumatology, University of Health Sciences, Umraniye Research and Training Hospital, Istanbul, Turkey; 5 Department of Pediatric Rheumatology, Faculty of Medicine, Istanbul University, Istanbul, Turkey; 6 Department of Pediatric Rheumatology, Faculty of Medicine, Dokuz Eylül University, Izmir, Turkey; 7 Department of Pediatric Rheumatology, Faculty of Medicine, Karadeniz Technical University, Trabzon, Turkey; 8 Department of Pediatric Rheumatology, Medicalpark Hospital, Gaziantep, Turkey; 9 Department of Pediatric Rheumatology, Istanbul Medeniyet University, Göztepe Prof. Dr. Süleyman Yalçın City Hospital, Istanbul, Turkey; 10 Department of Pediatric Rheumatology, Acıbadem Healthcare Group, Istanbul, Turkey; 11 Department of Pediatric Rheumatology, Gazi University Faculty of Medicine, Ankara, Turkey

**Keywords:** Juvenile, Idiopathic, Arthritis, Rheumatoid factor

## Abstract

**Objective:**

Rheumatoid factor (RF)-positive polyarthritis is the least common type of juvenile idiopathic arthritis (JIA). Functional disability in RF-positive polyarthritis patients is much more severe than in patients with other subtypes; but data on this subtype alone is limited. This study aimed to analyze clinical features, long-term follow-up, treatment response, and remission status in a large multicenter cohort of RF-positive polyarthritis patients.

**Methods:**

This retrospective study included RF-positive polyarthritis patients that were followed up for ≥ 6 months between 2017 and 2022 by the Pediatric Rheumatology Academy (PeRA)-Research Group (RG). Data on patient demographics, clinical and laboratory characteristics were obtained from medical charts. JIA treatments and duration of treatment were also recorded. The patients were divided into 2 groups based on methotrexate (MTX) response, as follows: group 1: MTX responsive, group 2: MTX unresponsive. Clinical and laboratory findings were compared between the 2 groups.

**Results:**

The study included 56 (45 female and 11 male) patients. The median age at onset of RF-positive polyarthritis was 13.2 years [(interquartile range) (IQR): 9.0–15.0 years] and the median duration of follow-up was 41.5 months (IQR: 19.5–75.7 months). Symmetrical arthritis affecting the metacarpophalangeal and proximal interphalangeal joints of the hands was commonly observed. Subcutaneous MTX was the preferred initial treatment; however, it was ineffective in 39 (69.6%) of the patients. Of 25 patients followed for 24 months, 56% still had active disease at 24 months.

**Conclusion:**

During 2 years of treatment, 44% of RF-positive polyarthritis patients have inactive disease, and they should be considered as a distinct and important clinical entity requiring aggressive and early treatment.

## Introduction

Juvenile idiopathic arthritis (JIA) is the most common chronic rheumatic disease in children [1, 2]. There are seven JIA subtypes according to the ILAR (International League of Associations for Rheumatology) classification system, each with a distinct epidemiological presentation, pathogenesis, genetic background, and clinical manifestations. The treatment approach, prognosis and associated morbidity vary according to JIA subtype [3–6]. Rheumatoid factor (RF)-positive polyarthritis is the least common JIA subtype with a frequency of 5% of JIA patients [2]. It is defined as involvement of ≥ 5 joints and by the presence of RF positivity on 2 occasions ≥ 3 months apart, both within the first 6 months following disease onset. As the disease may have a devastating course when not handled appropriately, the delay in diagnosis and treatment can be catastrophic by irreversible joint destruction as well as extra-articular complications [6, 7].

Among conventional disease-modifying anti-rheumatic drugs (DMARD), MTX is the most commonly used in pediatric rheumatology; however, there are only few evidence-based treatment guidelines for polyarticular JIA [8, 9]. Biological therapies, which were introduced after 2000, decreased both morbidity and the mortality rate in patients with rheumatic diseases [10, 11]. But despite these advances, a large number of JIA patients continue to have active disease in the long term, with or without damage. In all, 42%-67% of patients with JIA have active disease while transitioning to adult care, and 45%-50% have functional limitations [12–16].

Although remission and clinically inactive disease have been inconsistently defined in JIA, it is a known fact that patients with RF-positive polyarthritis have the lowest remission rates among JIA patients. Moreover, functional disability in RF-positive polyarthritis patients is much more severe than in patients with other subtypes; therefore, it is essential to evaluate disease activation, and articular and extra-articular damage during the disease course [15–18]. The literature contains limited data on the RF-positive polyarthritis subtype. Although several studies on JIA have been published, most provided general JIA data and didn’t concentrate on RF-positive polyarthritis as a distinct clinical entity. The present study aimed to analyze the demographic and clinical characteristics, treatment modalities, and response in thed long-term follow-up in a large multicentric cohort of RF-positive polyarthritis patients.

## Materials and methods

This multicenter, retrospective, cross-sectional, observational cohort study analyzed data from RF-positive polyarthritis patients followed at 10 pediatric rheumatology centers between 2017 and 2022 (Pediatric Rheumatology Academy [PeRA]-Research Group [RG]). The general methods of the PeRA-RG study have been described previously [19]. Inclusion criteria were diagnosis of RF-positive polyarthritis according to the ILAR criteria, and follow-up of ≥ 6 months. Data on patient gender and age, age at disease onset, and age at diagnosis were obtained from medical charts. An information form for collecting data on patients’ number of active joints, number of joints with limited motion, acute-phase reactant levels (erythrocyte sedimentation rate [ESR] and C-reactive protein [CRP]), RF titer and anti-cyclic citrullinated peptide (anti-CCP) levels, patient/parent VAS pain scores (0–10 cm) and physician VAS pain scores were completed at the time of diagnosis and throughout the follow-up period. JIA treatments and the duration of treatments were also recorded. Follow-ups were conducted at 3 months, 6 months, 12 months, 18 months, 24 months, and 36 months. JIA status was determined according to the Wallace et al. inactive disease is defined criteria system. Initially, all patients were treated with a 15 mg/m2/week subcutaneous injection of methotrexate. The patients were divided into 2 groups based on MTX response, as follows: group 1: MTX responsive group; group 2: MTX unresponsive group (MTX unresponsive group was defined as an active disease according to Wallace et al.criteria after the 3rd month of MTX treatment). Clinical and laboratory findings were compared between the 2 groups. Disease damage was measured using the Juvenile Arthritis Damage Index (JADI), which is composed of 2 parts 1 for the assessment of articular damage (JADI-A) and 1 for the assessment of extra-articular damage (JADI-E). The maximum JADI-A score is 72, and JADI-E is 17 [18–20]. The study protocol was approved by the hospital ethics committee.

### Statistical analysis

Statistical analysis was performed using IBM SPSS Statistics for Windows v.21.0 (IBM Corp., Armonk, NY). The study variables were investigated using visual (histograms and probability plots) and analytic methods (Kolmogorov–Smirnov and Shapiro–Wilk tests) to determine the normality of their distribution. Categorical parameters are presented as percentages. Parametric parameters are expressed as mean ± SD and non-parametric parameters are expressed as median (IQR). Categorical variables were compared using the chi-squared test or Fisher’s exact test, as appropriate. Differences in continuous data between the two groups were evaluated via the Student’s t-test or the Mann–Whitney U test, as appropriate. The Friedman test was used to compare the change in WBC, PLT, ESR, CRP, and VAS values, and the active joint counts between baseline, initiation of treatment, and after 6 months of treatment. The level of statistical significance was set at *P* < 0.05.

## Results

The study included 56 patients with RF-positive polyarthritis that were followed up between 2017 and 2022. Among the patients, 45 (80.4%) were female and 11 (19.6%) were male. The median age of the patients was 18.0 years (IQR: 6.0–25.0 years). The median age at symptom onset and diagnosis was 13.2 years (IQR: 9.0–15.0 years) and 13.9 years (IQR: 9.2–15.1 years), respectively. The median duration of follow-up was 41.5 months (IQR: 19.5–75.7 months) and the median duration from symptom onset to diagnosis was 4.0 months (IQR: 2.0–6.0 months). Baseline clinical and laboratory patient characteristics are given in Table 1. All patients (100%) were initially treated with MTX. The median duration of MTX treatment was 12 months (IQR: 3–120 months). Concomitantly with MTX, 47 (83.9%) patients received non-steroid anti-inflammatory drugs (NSAIDs) and 42 (75%) used oral corticosteroids. Four patients were required to switch MTX to leflunomide (LFN) due to gastrointestinal intolerance. However, none of the patients receiving LFN reached remission.

**Table 1:** The baseline and comparison of clinical and laboratory characteristics of patients

	**Whole group** ***n* = 56**	**Group 1** MTX responsive group ***n* = 17**	**Group 1** MTX unresponsive group ***n* = 39**	***p***
**Gender, female, n (%)**	45 (80.4%)	15 (88.2%)	30 (76.9%)	0.47
**Age at diagnosis (years), median (IQR)**	13.9 (9.2–15.1)	13.9 (9.7–15.0)	14.0 (9.0–15.4)	0.59
**Follow-up period (months), median (IQR)**	41.5 (19.5–75.7)	36 (10.5–85)	45 (24–72)	0.82
**Family history of rheumatic disease, n (%)**	12 (21.4%)	4 (23.5%)	8 (20.5%)	0.52
**Active joint count at the diagnosis, median (IQR)**	9 (6–15)	10 (7–15)	8 (6–16)	0.93
**Morning stiffness, n (%)**	51 (91.1%)	15 (88.2%)	36 (92.3)	0.63
**Number of joints with limited motion, median (IQR)**	1 (0–5)	1 (0–4)	1 (0–10)	0.57
**Physician VAS at the diagnosis, median (IQR)**	7 (5–8)	6 (5.5–8)	7 (5–8)	0.68
**Patient VAS at the diagnosis, median (IQR)**	7 (6–8)	7 (5–8)	8 (6–8)	0.53
**Laboratory findings at the diagnosis**				
** ESR, mm/hour, median (IQR)**	30.5 (16.2–49.7)	35 (8.5–47)	30 (17–58)	0.23
** CRP, mg/L, median (IQR)**	10.0 (2.7–18.7)	5.8 (2–15)	10.2 (3.8–20)	0.45
** The titer of RF at the diagnosis, median (IQR)**	105.1 (42.1–172.0)	47 (35–128)	114 (55.8–181)	0.22
** Anti-CCP positivity, n (%)**	38/46 (70.4%)	10/14 (71.4%)	28/32 (87.5%)	0.22
** HLA-B27 positivity, n (%)**	0/37 (0%)			
** ANA positivity, n (%)**	23 (41.1%)	7 (41.1%)	16 (41.0%)	1.00

Data expressed as median (IQR) (interquartile range) values

*ANA* Antinuclear antibody, *Anti-CCP* Anti- Cyclic Citrullinated Peptide, *CRP* C-reactive protein (mg/L, normal range 0–5), *ESR* Erythrocyte sedimentation rate (mm/h; normal range 0–20), *HLA-B27* Human leukocyte antigen-B27, *RF* Rheumatoid factor, *VAS* Visual analog scale (0–10 cm, 10 worst), *WBC* White blood cell (× 103/mm3)

In total, 17 (30.4%) patients achieved remission within a median of 4 months (IQR: 3–12 months), whereas MTX was ineffective in 39 (69.6%) patients. Among these 39 patients, 34 (60.7%) required a biological agent (BA) (5 patients’ parents refused biologic therapy, but continued oral corticosteroid therapy). Among these 34 patients the first-choice BA was as follows: etanercept (ETN): *n* = 22 (64.7%); adalimumab (ADA): *n* = 8 (23.5%); tocilizumab (TOC) *n* = 4 (11.8%) patients. In addition, 27 (79.4%) of these 34 patients achieved remission a median of 3 months (IQR: 1–4 months) after BA initiation. The initial BA was switched to another BA in 7 (20.6%) patients, as follows: initial BA (anti-TNF drug) to another BA (anti-TNF drug) of the same class: *n* = 2; initial BA to a BA with different mechanisms of action (TOC): *n* = 5.

The disease-related parameters during follow-up are summarized in Table 2. In all, 40 patients had a 12-month follow-up and 25 patients had a 24-month follow-up. Whereas, 42.9% of the patients who were followed up for 12 months had inactive disease, and 44% of the patients who were followed up for 24 months had inactive disease. There weren’t significant differences in age, gender, age at diagnosis, diagnostic delay, active joint count at diagnosis, patient/parent VAS pain scores at diagnosis, physician VAS pain score at diagnosis, acute-phase reactant levels (ESR and CRP) at diagnosis, or RF titer and anti-CCP levels between the patients with inactive disease and active disease at 24 months. Among the 9 patients that achieved inactive disease, treatment was ceased after a median of 20 months (IQR: 9–36 months) of inactive disease. In 4 of these 9 patients flare-ups occurred a median of 3 months (IQR: 3–5.2 months) after treatment cessation. Treatment was discontinued in 4 of 17 patients with MTX response. Flare occurred in 1 patient 6 months after treatment was discontinued.

**Table 2:** Disease-related parameters during the follow-up

**Parameters**	**3**^**rd**^** month** ***n* = 56**	**6**^**th**^** month** ***n* = 56**	**12**^**th**^** month** ***n* = 40**	**24**^**th**^** month** ***n* = 25**	**36**^**th**^** month** ***n* = 10**
**Active joint count**^a^	2 (0–20)	1 (0–18)	0 (0–12)	0 (0–12)	0 (0–10)
**Number of joints with limited motion**^a^	0 (0–20)	0 (0–19)	0 (0–10)	0 (0–12)	0 (0–8)
**Patient VAS**^a^	5 (0–8)	0 (0–7)	2 (0–10)	0 (0–10)	0 (0–10)
**Physician VAS**^a^	4 (0–8)	0 (0–7)	2 (0–10)	0 (0–10)	3 (1–10)
**ESR, mm/hour**^a^	13 (2–70)	12 (2–93)	11.5 (2–46)	10 (2–41)	13 (5–110)
**CRP, mg/L**^a^	1.9 (1–33)	1.4 (1–66)	1.8 (1–40)	1.4 (1–15.6)	3.3 (2–186)
**JADI-A**^a^	0 (0–16)	0 (0–10)	0 (0–6)	0 (0–8)	0 (0–6)
**JADI-E**^a^	0 (0–4)	0 (0–4)	0 (0–4)	0 (0–4)	0 (0–2)
**Inactive disease, n (%)**	17 (30.3%)	34 (60.7%)	24 (60%)	11 (44%)	2 (20%)
**Active disease, n (%)**	39 (69.7%)	22 (39.3%)	16 (40%)	14 (56%)	8 (80%)

*Anti-CCP* Anti- Cyclic Citrullinated Peptide, *CRP* C-reactive protein (mg/L, normal range 0–5), *ESR* Erythrocyte sedimentation rate (mm/h; normal range 0–20), *RF* Rheumatoid factor, *VAS* Visual analog scale (0–10 cm, 10 worst), *JADI-A* Juvenile Arthritis Damage Index articular damage, *JADI-E* Juvenile Arthritis Damage Index extraarticular damage

^a^Data expressed as median (IQR) values

**Fig. 1:**
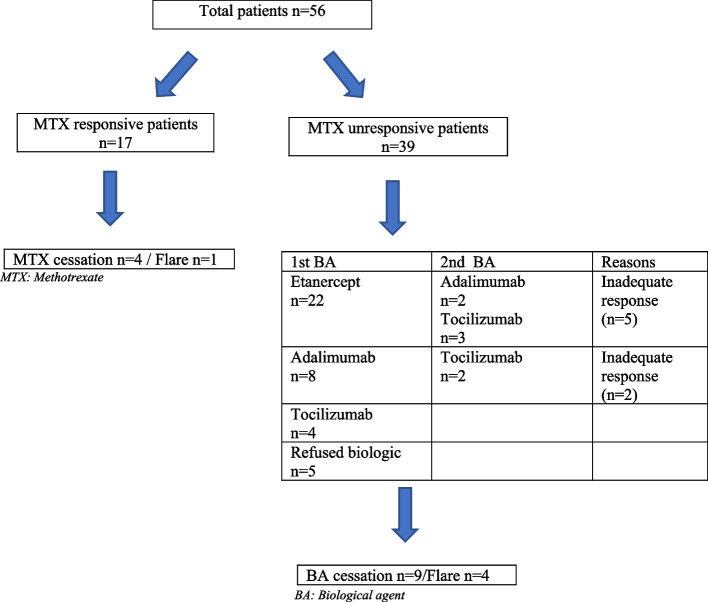
The treatment of the patients

The treatment is schematized summarized in Fig. 1.

There were no significant differences in age, gender, age at diagnosis, antinuclear antibody (ANA) positivity, the active joint count at diagnosis, patient/parent VAS pain scores at diagnosis, physician VAS pain score at diagnosis, acute-phase reactant levels (ESR and CRP) at diagnosis, or RF titer and anti-CCP levels between the patients that did and did not respond to MTX (Comparison of clinical and laboratory characteristics of between the 2 groups are given Table 1). Furthermore, there were no significant differences between the patients that did and did not respond to MTX in the JADI-A and JADI-E scores at the 6-, 12-, and 24-month follow-up.

## Discussion

The data regarding the outcome of JIA is increasing worldwide; however, published data on the outcome of RF-positive polyarthritis remain scarce. Although advances in JIA treatment have led to a reduction in disease-related joint damage and an increase in physical function and quality of life, some patients with RF-positive polyarthritis still seem to be in active disease. In the present study, 44% of RF-positive polyarthritis patients had inactive disease at the 24-month follow-up.

The median age of onset of RF-positive polyarthritis is 9–11 years (range: 1.5–15 years) and affected females outnumber males (from 4:1–13:1) in large series [21–23]. Consistent with the literature, in the present study, the median age at diagnosis was 13.2 years and female predominance was noted (4:1).

It was previously noted that the upper and lower extremity large and small joints are affected, as well as the cervical spine and temporomandibular joint (TMJ), whereas the thoracic and lumbar spine and sacroiliac joints are spared. Although large joints are commonly involved, the characteristic pattern is symmetrical arthritis affecting the metacarpophalangeal (MCP) and proximal interphalangeal (PIP) joints of the hands, the wrists, and the metatarsophalangeal (MTP) and PIP joints of the feet [3, 23]. Similarly, in the present study, symmetrical arthritis affecting the MCP and PIP joints of the hands were commonly observed (82%). Compared to RF-negative polyarthritis, TMJ involvement is less common, but can occur in up to 30% of RF-positive polyarthritis patients [24, 25]. In the present cohort hip involvement was noted in 6 (10.7%) patients, cervical spine involvement was noted in 5 (8.9%) patients, and TMJ involvement was noted in 3 (5.3%) patients. Furthermore, the sternoclavicular joint was affected in 1 patient, the sacroiliac joint was affected in 1 patient, and the coxofemoral joint was affected in 1 patient. Subcutaneous nodules, uveitis, and other extraarticular disease manifestations of the disease were not observed in the present cohort.

Among all RF-positive polyarthritis patients, 42%-56% have ANA positivity [2]. Similarly in the present study, ANA positivity was noted in 41.1% of the patients. In the literature, the frequency of ACPA in RF-positive polyarthritis patients varies from 57 to 90%. ACPA correlates with disease severity and joint damage evidenced by radiographs, [16, 26, 27]; however, in the present study ACPA was not associated with disease activity, estimation of MTX response, or JADI scores. This may be due to the fact that ACPA was not studied in all patients.

In 2005 Viola et al. [18] studied 158 JİA patients with a mean follow-up period of 7.3 years, and reported a median JADI-A score of 0 (IQR: 0–39) and median JADI-E score of 0 (IQR: 0–7)). Subsequently, Menon et al. [17] studied patients JIA with a mean follow-up of 2 years, reporting a median JADI-A score of 0 (IQR: 0–52) and a median JADI-E score of 0 (IQR: 0–6). In the present study the maximum scores were be found lower than those that were previously reported. This may be due to the increased use of biologic drugs in recent years.

Current treatment recommendations for RF-positive polyarthritis MTX treatment should be initiated at the time of diagnosis unless contraindicated [28]. Persistent high or moderate disease activity despite MTX treatment necessitates prompt switch to biological therapy [28–30]. In one study, patients with RF-positive polyarthritis had the lowest drug-free remission rate among children with chronic arthritis [13]. Therefore, early aggressive treatment has long been accepted for RF-positive polyarthritis. In this study, all patients received MTX as first-line therapy, but 60.7% of the patients did not achieve remission with MTX and biologics were added to their treatment. There has been a rapid expansion of biologic therapies that effectively treat JIA, including RF-positive polyarthritis. The first biologic DMARD studied in JIA patients was etanercept. The efficacy and acceptable safety profile of etanercept was demonstrated in a randomized controlled trial (RCT) published in 2000 that included patients with polyarticular JIA that were resistant or intolerant to MTX [31]. Over the following years other anti-TNF inhibitors, including adalimumab, infliximab, and golimumab, the IL-6 inhibitor tocilizumab, and costimulatory disruption abatacept were tested in RCTs that included patients with polyarticular JIA [32–34]. These approaches were reported to result in clinically inactive disease in a significant proportion of polyarticular JIA patients [11, 30–34]. In the present study, 34 (60.7%) of the patients were treated with a BA, of which 25 were followed-up for 24 months; however, (*n* = 14) 56% still had active disease at 24 months. In all, only 44% of our cohort achieved remission during the 24-month follow-up, suggesting that the window of opportunity for early intervention might be missed. Additional larger-scale international studies are needed to predict non-responsiveness to MTX in RF-positive polyarthritis patients more accurately.

Although this study is limited in its retrospective design and small sample, to our knowledge it is the largest RF-positive polyarthritis series of children in Turkey evaluating the remission status as a real-life data. Even so, considering the limited number of studies on RF-positive polyarthritis, we believe that our study, which was carried out at the largest pediatric rheumatology clinics in Turkey, is valuable.

## Conclusion

Not all RF-positive polyarthritis patients treated with MTX are going to achieve remission and there is unfortunately no known way to predict MTX resistance in this group of patients; this may be due to the small number of patients. But currently in the literature there does not appear to be a marker to predict MTX resistance.The present findings indicate the importance of developing targeted therapy strategies and identifying parameters that can predict MTX resistance in patients with RF-positive polyarthritis. We suggest that RF positive JIA be considered as a separate entity.

## Data Availability

The data sets used and analysed during the current study are available from the corresponding author on reasonable request. No information (including demographic data, initials, date of birth), that would enable identification of any patient, were recorded or requested to maintain subject confidentiality; only the patient ages were collected. Examinations, diagnostic measures, findings and observations routinely performed in patients included in this retrospective study were entered according to the research plan.

## Abbreviations

RF: Rheumatoid factor
JIA: Juvenile idiopathic arthritis
MTX: Methotrexate
ILAR: International League of Associations for Rheumatology
DMARD: Disease-modifying anti-rheumatic drugs
IQR: Interquartile range
PeRA-RG: Pediatric Rheumatology Academy Research Group
ESR: Erythrocyte sedimentation rate
CRP: C-reactive protein
CCP: Cyclic citrullinated peptide
JADI: Juvenile Arthritis Damage Index
JADI-A: Juvenile Arthritis Damage Index- articular damage
JADI-E: Juvenile Arthritis Damage Index- extra-articular damage
NSAIDs: Non-steroid anti-inflammatory drugs
LFN: Leflunomide
BA: Biological agent
ETN: Etanercept
ADA: Adalimumab
TOC: Tocilizumab
TMJ: Temporomandibular joint
MCP: Metacarpophalangeal
PIP: Proximal interphalangeal
MTP: Metatarsophalangeal

## References

[1] Martini A, Ravelli A, Avcin T, Beresford MW, Burgos-Vargas R, Cuttica R (2019). Toward New classification criteria for juvenile idiopathic arthritis: first steps, pediatric rheumatology international trials organization international consensus. J Rheumatol.

[2] Petty RE, Southwood TR, Manners P, Baum J, Glass DN, Goldenberg J (2004). International league of associations for rheumatology classification of juvenile idiopathic arthritis: second revision, Edmonton, 2001. J Rheumatol.

[3] Helmick CG, Felson DT, Lawrence RC, Gabriel S, Hirsch R, Kwoth CK (2008). Estimates of the prevalence of arthritis and other rheumatic conditions in the United States. Part I. Arthritis Rheum.

[4] Ravelli A, Martini A (2007). Juvenile idiopathic arthritis. Lancet.

[5] Saurenmann RK, Rose JB, Tyrrell P, Feldman BM, Laxer RM, Schneider R (2007). Epidemiology of juvenile idiopathic arthritis in a multiethnic cohort: ethnicity as a risk factor. Arthritis Rheum.

[6] Duffy CM, Colbert RA, Laxer RM, Schanberg LE, Bowyer SL (2005). Nomenclature and classification in chronic childhood arthritis. Arthritis Rheum.

[7] Ringold S, Angeles-Han ST, Beukelman T, Lovell D, Cuello CA, Becker ML (2019). 2019 American College of Rheumatology/Arthritis foundation guideline for the treatment of juvenile idiopathic arthritis: therapeutic approaches for non-systemic polyarthritis, sacroiliitis, and enthesitis. Arthritis Rheum.

[8] Lovell DJ, Giannini EH, Reiff A, Cawkwell GD, Silverman ED, Nocton JJ (2000). Etanercept in children with Polyarticular juvenile rheumatoid Arthritis. N Engl J Med.

[9] Ruperto N, Lovell DJ, Cuttica R, Wilkinson N, Woo P, Espada G (2007). A randomized, placebo-controlled trial of infliximab plus methotrexate for the treatment of polyarticular-course juvenile rheumatoid arthritis. Arthritis Rheum.

[10] Guzman J, Oen K, Tucker LB, Huber AM, Shiff N, Boire G (2015). The outcomes of juvenile idiopathic arthritis in children managed with contemporary treatments: results from the ReACCh-out cohort. Ann Rheum Dis.

[11] Giancane G, Muratore V, Marzetti V, Quilis N, Benavente BS, Bagnasco F (2019). Disease activity and damage in juvenile idiopathic arthritis: methotrexate era versus biologic era. Arthritis Res Ther.

[12] Glerup M, Herlin T, Twilt M (2017). Clinical outcome and long-term remission in JIA. Curr Rheumatol Rep.

[13] Wallace CA, Huang B, Bandeira M, Ravelli A, Giannini EH (2005). Patterns of clinical remission in select categories of juvenile idiopathic arthritis. Arthritis Rheum.

[14] Ravelli A (2004). Toward an understanding of the long-term outcome of juvenile idiopathic arthritis. Clin Exp Rheumatol.

[15] Oen K, Reed M, Malleson PN, Cabral DA, Petty RE, Rosenberg AM (2003). Radiologic outcome and its relationship to functional disability in juvenile rheumatoid arthritis. J Rheumatol.

[16] van Rossum M, van Soesbergen R, de Kort S, ten Cate R, Zwinderman AH, de Jong B (2003). Anti-cyclic citrullinated peptide (anti-CCP) antibodies in children with juvenile idiopathic arthritis. J Rheumatol.

[17] Menon NVB, Peethambaran G, Puthiyapurayil AT, Nambudakath C, Arakkal R (2018). Clinical profile and juvenile arthritis damage index in children with juvenile idiopathic arthritis: a study from a tertiary care center in south India. Int J Rheum Dis.

[18] Viola S, Felici E, Magni-Manzoni S, Pistorio A, Buoncompagni A, Ruperto N (2005). Development and validation of a clinical index for assessment of long-term damage in juvenile idiopathic arthritis. Arthritis Rheum.

[19] Sözeri B, Sonmez HE, Demir F, Cakan M, Ozturk K, Ozdel S (2021). Time to collaborate: objectives, design, and methodology of PeRA-research group. North Clin Istanb.

[20] Consolaro A, Giancane G, Ravelli A, Petty RE, Laxer RM, Lindsley CB, Wedderbun LR, Mellins ED, Fuhlbrigge RR (2021). Clinical Outcome Measures in Pediatric Rheumatic Diseases. Textbook of pediatric rheumatology.

[21] Fujikawa S, Okuni M (1997). Clinical analysis of 570 cases with juvenile rheumatoid arthritis: results of a nationwide retrospective survey in Japan. Acta Paediatr Jpn.

[22] Bowyer S, Roettcher P (1996). Pediatric rheumatology clinic populations in the United States: results of a 3 year survey. Pediatric Rheumatology Database Research Group. J Rheumatol.

[23] Denardo BA, Tucker LB, Miller LC, Szer IS, Schaller JG (1994). Demography of a regional pediatric rheumatology patient population. Affiliated Children’s Arthritis Centers of New England. J Rheumatol.

[24] Twilt M, Mobers SM, Arends LR, ten Cate R, van Suijlekom-Smit L (2004). Temporomandibular involvement in juvenile idiopathic arthritis. J Rheumatol.

[25] Twilt M, Schulten AJM, Verschure F, Wisse L, Prahl-Andersen B, van Sujilekom-Smit LWA (2008). Long-term follow-up of temporomandibular involvement in juvenile idiopathic arthritis. Arthritis Rheum.

[26] Majka DS, Deane KD, Parrish LA, Lazer AA, Baron AE, Walker CW (2008). Duration of preclinical rheumatoid arthritis-related autoantibody positivity increases in subjects with older age at time of disease diagnosis. Ann Rheum Dis.

[27] Nielen MMJ, van Schaardenburg D, Reesink HW, van de Stadt RJ, van der Horst-Bruinsma IE, de Koning MHMT (2004). Specific autoantibodies precede the symptoms of rheumatoid arthritis: a study of serial measurements in blood donors. Arthritis Rheum.

[28] Beukelman T, Patkar NM, Saag KG, Tolleson-Rinehart S, Cron RQ, Dewitt EM (2011). 2011 American College of Rheumatology recommendations for the treatment of juvenile idiopathic arthritis: initiation and safety monitoring of therapeutic agents for the treatment of arthritis and systemic features. Arthritis Care Res (Hoboken).

[29] Stoll ML, Cron RQ (2013). Treatment of juvenile idiopathic arthritis in the biologic age. Rheum Dis Clin North Am.

[30] Tynjala P, Vahasalo P, Tarkiainen M, Kröger L, Aalto K, Malin M (2011). Aggressive combination drug therapy in very early polyarticular juvenile idiopathic arthritis (ACUTE-JIA): a multicentre randomised open-label clinical trial. Ann Rheum Dis.

[31] Lovell DJ, Ruperto N, Goodman S, Reiff A, Jung L, Jarosova K (2008). Adalimumab with or without methotrexate in juvenile rheumatoid arthritis. N Engl J Med.

[32] Ruperto N, Lovell DJ, Quartier P, Paz E, Rubio-Perez N, Silva CA (2008). Abatacept in children with juvenile idiopathic arthritis: a randomised, double-blind, placebo-controlled withdrawal trial. Lancet.

[33] Wallace CA, Giannini EH, Spalding SJ, Hashkes PJ, O’Neil KM, Zeft AS (2012). Trial of early aggressive therapy in polyarticular juvenile idiopathic arthritis. Arthritis Rheum.

[34] Wallace CA, Giannini EH, Huang B, Itert L, Ruperto N, Childhood Arthritis Rheumatology Research Alliance; Pediatric Rheumatology Collaborative Study Group; Paediatric Rheumatology International Trials Organisation (2011). American college of Rheumatology provisional criteria for defining clinical inactive disease in select categories of juvenile idiopathic arthritis. Arthritis Care Res.

